# Sirolimus-Induced Hepatitis in Two Patients with Hyperinsulinemic Hypoglycemia

**DOI:** 10.4274/jcrpe.5335

**Published:** 2018-07-31

**Authors:** Belma Haliloğlu, Heybet Tüzün, Sarah E. Flanagan, Muhittin Çelik, Avni Kaya, Sian Ellard, Mehmet Nuri Özbek

**Affiliations:** 1 Yeditepe University Faculty of Medicine, Department of Pediatric Endocrinology, İstanbul, Turkey; 2 Diyarbakır Child Health Hospital, Clinic of Neonatology, Diyarbakır, Turkey; 3 University of Exeter Medical School, Institute of Biomedical and Clinical Science, Exeter, United Kingdom; 4 University of Health Sciences, Diyarbakır Gazi Yaşargil Training and Research Hospital, Clinic of Pediatric Endocrinology, Diyarbakır, Turkey

**Keywords:** Hyperinsulinemic hypoglycemia, sirolimus, hepatitis, liver enzymes

## Abstract

Sirolimus has been reported to be effective in the treatment of the diffuse form of congenital hyperinsulinism (CHI), unresponsive to diazoxide and octreotide, without causing severe side effects. Two newborns with CHI due to homozygous ABCC8 gene mutations were started on sirolimus aged 21 and 17 days, due to lack of response to medical treatment. A good response to sirolimus was observed. At follow-up after ten and two months of treatment, liver enzymes were found to be increased [serum sirolimus level 1.4 ng/mL (normal range: 5-15), aspartate aminotransferase (AST): 298U/L, alanine aminotransferase (ALT): 302U/L and serum sirolimus level: 9.9 ng/mL, AST: 261U/L, ALT: 275U/L, respectively]. In Case 1, discontinuation of the drug resulted in normalization of liver enzymes within three days. Two days after normalization, sirolimus was restarted at a lower dose, which resulted in a repeated increase in transferases. In Case 2, a reduction of sirolimus dose caused normalization of liver enzymes within ten days. When the dose was increased, enzymes increased within three days. Sirolimus was discontinued in both cases.

The rapid normalization of liver enzyme levels after sirolimus withdrawal or dose reduction; elevation of transaminases after restart or dose increase and rapid normalization after sirolimus withdrawal were findings strongly suggestive of sirolimus-induced hepatitis.

To the best of our knowledge, this is the first report of sirolimus-induced hepatitis in CHI. Sirolimus is a promising drug for CHI patients who are unresponsive to medical treatment, but physicians should be vigilant for adverse effects on liver function.

## What is already known on this topic?

Sirolimus is an alternative for the treatment of congenital hyperinsulinism unresponsive to diazoxide and octreotide.

## What this study adds?

This is the first report of sirolimus-induced hepatitis in pediatric patients with hyperinsulinemic hypoglycemia.

## Introduction

Congenital hyperinsulinism (CHI) is characterized by inappropriate insulin secretion despite hypoglycemia. It is a heterogeneous disorder with the clinical manifestations ranging from severe hypoglycemia in the newborn period to mild hypoglycemia in childhood (1,2). The incidence is approximately 1:30.000 live births but is increased in populations with a high prevalence of consanguinity (3). Most cases of CHI are caused by autosomal recessive mutations in the *ABCC8* and *KCNJ11* genes (1).

Historically the treatment of severe, diffuse CHI, unresponsive to diazoxide and octreotide was subtotal pancreatectomy. This surgery has been associated with a high incidence of insulin-dependent diabetes, persistent hypoglycemia and exocrine pancreatic insufficiency (4). As a novel agent, the mammalian target of rapamycin (mTOR) inhibitor, sirolimus, has been recommended for the treatment of the diffuse form of CHI, unresponsive to diazoxide and octreotide. It has been reported to be a safe agent in pediatric cases (5,6,7). Herein, we report two cases of diazoxide and octreotide unresponsive CHI, due to homozygous *ABCC8* gene mutations in which sirolimus had to be discontinued because of drug related hepatotoxicity.

## Case Reports

### Case 1

A female infant presented with severe hypoglycemia on the first day of life. CHI was diagnosed based on laboratory findings. She was normoglycemic with intravenous (iv) glucose, diazoxide, iv glucagon and octreotide on day 16 but the reduction in glucose requirement was not successful during the next five days (Table 1). She also had congenital hypothyroidism with normal thyroid ultasonography (TSH: >100 uIU/mL, sT4:0.7 ng/dL) and was euthyroid with L-thyroxine (12 mcg/kg/day).

18F-DOPA positron emission tomography/computed tomography (PET/CT) scanning could not be performed but sequence analysis identified a novel homozygous p.H59P (c.176A>C) missense mutation in the proband’s *ABCC8* gene. *In silico* analysis predicted the variant was likely to be pathogenic and that the affected residue was highly conserved across species (Alamut, Rouen, France). The identification of a recessively inherited ABCC8 mutation in the patient was consistent with diffuse pancreatic disease. After consent from the parents, sirolimus was started at a dose of 0.5 mg/m^2^/day on day 21. The serum level of sirolimus and laboratory tests (full blood count, kidney and liver function tests, lipid profile, electrolytes) were checked every five days, to maintain the serum sirolimus concentration between 5-15 ng/dL. The patient was discharged on day 72 with oral feeding, subcutaneous octreotide (40 mcg/kg/d) and oral sirolimus (3 mg/m^2^/day). The sirolimus level and biochemical markers were checked at monthly intervals.

**Figure 1:**
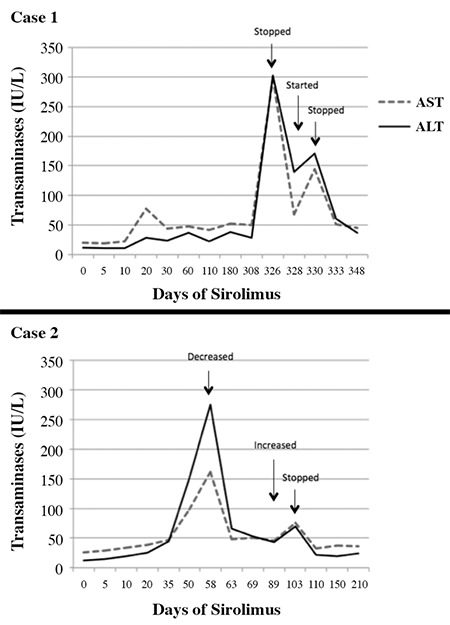
Liver enzyme levels (AST: aspartate aminotransferase, ALT: alanine aminotransferase) of Case 1 and Case 2 during sirolimus treatment and immediately after cessation

Since she was normoglycemic, the octreotide dose was decreased during follow-up. At the age of 10 months the patient presented with diarrhea. At this time, she was being treated with octreotide (6 mcg/kg/d) and sirolimus (3.1 mg/m^2^/day) and was normoglycemic. Her laboratory tests revealed elevated liver enzymes (Table 2). The coagulation tests, bilirubin levels, alkaline phosphatase (ALP), gamma-glutamyl transpeptidase (GGT) and abdominal ultrasound results were all normal. Although the sirolimus level was below the therapeutic range (1.4 ng/mL), it was discontinued due to its known hepatotoxic effect. The liver enzyme levels during dose adjustments are shown in the Figure 1. After sirolimus was discontinued, the octreotide dose was increased to 45 mcg/kg/d to achieve normoglycemia and four months later the patient was switched to octreotide-long-acting release (LAR). She is currently 18 months of age with normal neuromotor development and normoglycemia, treated solely with octreotide-LAR (15 mg/monthly, 41 mcg/kg/d) and oral feedings with three hourly intervals. Her most recent HbA1c level was 4.9% (30 mmol/mol) and also, she is euthyroid on L-thyroxine treatment.

### Case 2

This female infant was referred to our clinic on day 14 of life with CHI resistant to medical therapy (Table 1) and the reduction in glucose requirement was not successful. She had also congenital hypothyroidism with normal thyroid ultrasound (TSH: >100 uIU/mL, sT4:0.9 ng/dL) and was euthyroid with L-thyroxine (8 mcg/kg/d).

Sequence analysis identified a previously reported homozygous missense mutation, p.A1185E (c.3554C>A), in ABCC8 (8). The presence of a homozygous mutation in the patient was in keeping with diffuse pancreatic disease. After consent from the parents was obtained, sirolimus (0.5 mg/m^2^/day) was added, due to no reduction in the glucose requirement by day 17. Serum levels of sirolimus were checked every five days to maintain a therapeutic serum level as before. Neither clinical nor laboratory side effects were observed. She was discharged at age 40 days with sirolimus 0.4 mg/m^2^/day and octreotide 23 mcg/kg/d.

One month later, routine blood tests for side effects revealed elevated liver enzymes (Table 2) without any clinical symptoms. Sirolimus level at this time was 9.9 ng/mL in the middle of the therapeutic range. All other laboratory tests (blood count, kidney function tests, ALP, GGT, bilirubin levels) and abdominal ultrasound revealed normal results. The liver enzyme levels during dose adjustments are shown in Figure 1. As sirolimus was discontinued, the dose of octreotide was increased from 10 to 45 mcg/kg/d. Although, the glucose levels were generally close to the lower limit of normal, with frequent oral feedings and applying a maximum dose of octreotide, we were able to protect the patient from severe hypoglycemia (a glucose level <50 mg/dL). Subcutaneous octreotide was switched to octreotide-LAR five months later. The patient is currently 13 months of age and normoglycemic with octreotide-LAR (15 mg/monthly, 45 mcg/kg/d) and oral feedings at 4 hours intervals. Last HbA1c is 4.2% (22 mmol/mol) and also, she is euthyroid on L-thyroxine treatment.

## Discussion

The aim of treatment in CHI is to achieve normoglycemia and to prevent neurological damage. However, the clinical management of severe, diffuse CHI, unresponsive to medical treatment is still a vexing clinical problem (4). In a recent study, mTOR inhibitor, sirolimus, has been reported to be a novel agent for the treatment of diazoxide unresponsive CHI. Therapy with sirolimus achieved normoglycemia with no major adverse effect in four cases (5). We now report two further cases with severe CHI due to a homozygous ABCC8 mutation. Both were successfully treated with sirolimus consistent with previous reports, but sirolimus had to be discontinued because of drug-induced hepatitis.

In adult studies, various side effects of mTOR inhibitors have been reported which include bone marrow suppression, dyslipidemia, immunosuppression, elevation of liver enzymes, renal dysfunction, pneumonitis and stomatitis. These were reversible with dose reduction (9,10). In children, this drug was reported to be well tolerated in several studies with normal or high doses (1-6 mg/m^2^/d) (11,12,13). The main side effect reported in these studies was oral mucositis. However, in a recent study, Szymanowski et al (14) investigated the efficacy and adverse effect profile of sirolimus in the treatment of severe CHI. These authors detected adverse events such as hypertriglyceridemia, anemia, stomatitis, sepsis, varicella zoster and gut dysmotility in 80% of their patients, but also reported a 30% therapeutic success rate.

Hepatotoxicity is another known side effect of sirolimus, resulting in transient and mild increase in liver enzymes. Its incidence was reported to be 17% in patients with renal transplant (15). Senniappean et al (5) and Méder et al (6) reported mild, transient elevation of liver enzyme concentrations. These increases were less than double the normal range and resolved spontaneously or with reduction in sirolimus dose (5,6). Although sirolimus appears to be safe in terms of hepatotoxicity, cases with severe sirolimus-induced hepatitis have been reported. One report was that of a patient with renal transplantation who received sirolimus as an initial immunosuppressive in the post-transplant period (16). At the 16^th^ month post-transplant, increased liver enzyme levels were detected [maximum aspartate aminotransferase (AST): 368 IU/L, alanine aminotransferase (ALT): 579 IU/L] with a serum sirolimus level of 6.3 ng/dL. After sirolimus withdrawal, quick normalization of aminotransferases was observed. Jacques et al (17) reported another case with renal transplantation. In the second month of sirolimus, biochemical tests showed acute hepatitis (AST: 861 IU/l, ALT: 609 IU/L) with signs of hepatic insufficiency. The serologic and autoimmune markers for hepatitis were normal. Despite a normal sirolimus level (10 ng/mL), it was withdrawn and transaminase levels normalized within five weeks. In our two cases, after sirolimus was discontinued in one case and decreased in the other, the normalization of transaminases was observed within a few days.

While octreotide is usually well tolerated in most patients with CHI, octreotide induced hepatitis has been reported in a few patients (18,19,20,21). Hepatitis was found to develop even with doses within the normal range, but the withdrawal of octreotide resulted in resolution. The rapid normalization of liver enzyme levels after sirolimus withdrawal and dose reduction, in our first and second case respectively, followed by elevation of transaminases after restart or dose increase and rapid normalization after sirolimus was again withdrawn while the patient continued with octreotide treatment provides robust evidence of sirolimus-induced hepatitis.

Fortunately, both patients are now normoglycemic with octreotide-LAR and frequent feedings. This observation suggests that this entity may tend to become milder over time. It also suggests that a good response to octreotide-LAR may be expected as the patients get older.

Octreotide may affect thyroid hormones and may cause hypothyroidism with a concomitant low TSH level. However, hypothyroidism with elevated TSH levels was also reported in two cases with octreotide treated CHI due to *ABCC8* gene mutation (19,20). Similarly, our cases had elevated TSH levels with a low free thyroxine that is a characteristic finding for primary hypothyroidism. This is most probably a coincidental finding since patients on octreotide therapy usually develop central hypothyroidism marked by low TSH. Further tests will be done for the etiology of primary hypothyroidism in later years.

In conclusion, sirolimus is a promising drug for diazoxide and octreotide unresponsive CHI patients, but physicians should be vigilant for its adverse effects which may necessitate the withdrawal of the drug.

## Figures and Tables

**Table 1:**
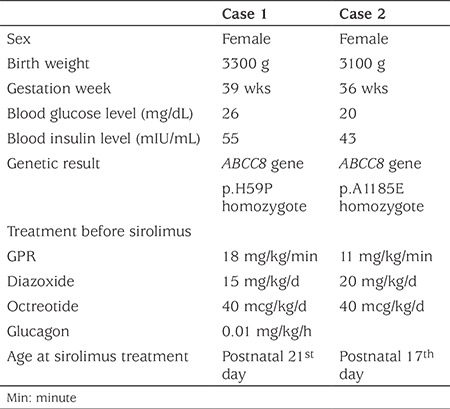
The clinical features of the patients before sirolimus

**Table 2:**
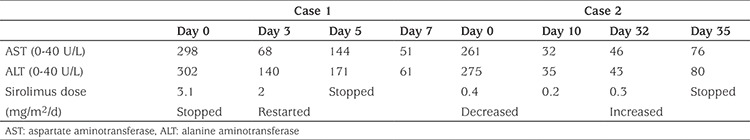
The liver enzyme levels of the patients during hepatotoxic period of sirolimus
